# Plasma-Functionalized Liquids for Decontamination of Viable Tissues: A Comparative Approach

**DOI:** 10.3390/ijms251910791

**Published:** 2024-10-08

**Authors:** Alexander Pogoda, Yuanyuan Pan, Monika Röntgen, Sybille Hasse

**Affiliations:** 1 Department of Plasma Life Science, Leibniz Institute for Plasma Science and Technology e.V. (INP), 17489 Greifswald, Germany; alexander.pogoda@inp-greifswald.de (A.P.); yuanyuan.pan@inp-greifswald.de (Y.P.); 2 Working Group Cell Biology of Muscle Growth, Research Institute of Farm Animal Biology (FBN), 18196 Dummerstorf, Germany; roentgen@fbn-dummerstorf.de

**Keywords:** plasma-functionalized liquid, antimicrobial activity, plasma sources, RONS, stem cells, cultured meat

## Abstract

Plasma-functionalized liquids (PFLs) are rich in chemical species, such as ozone, hydrogen peroxide, singlet oxygen, hydroxyl radical and nitrogen oxides, commonly referred to as reactive oxygen and nitrogen species (RONS). Therefore, manifold applications are being investigated for their use in medicine, agriculture, and the environment. Depending on the goal, a suitable plasma source concept for the generation of PFLs has to be determined because the plasma generation setup determines the composition of reactive species. This study investigates three PFL-generating plasma sources—two spark discharges and a flow dielectric barrier discharge (DBD) system—for their efficacy in eliminating microbial contaminants from tissue samples aiming to replace antibiotics in the rinsing process. The final goal is to use these tissues as a cell source for cell-based meat production in bioreactors and thereby completely avoid antibiotics. Initially, a physicochemical characterization was conducted to better understand the decontamination capabilities of PFLs and their potential impact on tissue viability. The results indicate that the flow DBD system demonstrated the highest antimicrobial efficacy due to its elevated reactive species output and the possibility of direct treatment of tissues while tissue integrity remained. Achieving a balance between effective large-scale decontamination and the biocompatibility of PFLs remains a critical challenge.

## 1. Introduction

Plasma-functionalized liquids (PFLs) are liquids that have been enriched with reactive oxygen and nitrogen species (RONS) during a plasma process at ambient conditions. Generated primary RONS dissolve in liquid, interact with the liquid and with each other, and may form other more stable compounds (secondary RONS). Generally, there are three typical approaches to generate bulk PFLs: one takes advantage of the direct discharge in liquids, the second employs plasma gas–liquid interactions, while the third utilizes bubbles inside liquids for multiphase discharges [1,2].

One main characteristic of PFLs is the antimicrobial activity due to the synergistic effects of nitrate (NO_3_^−^), nitrite (NO_2_^−^), and hydrogen peroxides (H_2_O_2_) combined with an acidic pH [3,4,5]. These PFLs contain multiple highly reactive oxygen and nitrogen species (e.g., H_2_O_2_, ozone (O_3_), NO_2_^−^, NO_3_^−^, hydroxyl radical (•OH), singlet oxygen (^1^O_2_), superoxide anion radical (•O_2_^−^), nitric oxide radical (•NO), peroxynitrous acid (ONOOH), and peroxynitrate (OONOOH)) that target a broad spectrum of microbial strains and act at multiple microbial sites [3,6,7,8,9,10]. Development of microbial resistances has not been observed so far due to the physicochemical mode of action [11]. Hence, over recent years, a broad range of applications has emerged that include food decontamination of vegetables [12], seeds [13], and other foods (e.g., meat and fish) [1,13,14]. Beyond food safety, PFLs have also shown promising effects in medical sterilization and wound healing, where their antimicrobial properties are used to disinfect surfaces and promote tissue recovery [15]. Moreover, PFLs are being explored for environmental remediation, including wastewater treatment and pollutant degradation [16,17,18,19].

Based on these successful applications, the use of PFLs for decontaminating animal-derived tissue samples seems a promising novel approach to extend biotechnological applications. In particular, umbilical cords from farm animals, such as pigs (porcine umbilical cords, pUCs), contain valuable pluripotent mesenchymal stem cells (MSCs) that are suitable as starting material for the production of cultured meat [15,16,17] as an ethical and sustainable source of animal protein. However, a critical step before isolating primary stem cells from the tissue is the effective rinsing of pUCs to ensure sterility and avoid contaminants. Classically, antibiotics are used for rinsing animal-derived tissues. Concerns regarding resistance to antibiotics and the negative environmental impact necessitate the exploration of alternative rinsing strategies that align with sustainability and ethical cellular agriculture principles [20]. Furthermore, antibiotic residues may be present in the final cell-based food product and can present a health hazard equivalent to conventional meat products.

PFLs are recognized for their environmentally friendly and sustainable properties. However, scalability of PFL-producing systems for industrial-level decontamination remains challenging in many aspects, such as energy efficiency, reactor design, control of reactive species, cost, and infrastructure or process time, and throughput [21,22]. These challenges must be addressed through optimized system design, improved energy management, and careful regulation of reactive species in large-scale applications. This study aimed at investigating innovative rinsing alternatives for pUCs to develop a sustainable pathway for harvesting stem cells for subsequent cultured meat production. We explored the potential of PFLs mediated by different plasma sources: wINPlas, wINPlas XXL, and a flow through a dielectric barrier discharge (DBD) system. The effectiveness of these PFLs in eliminating microbial contaminants from pUCs was evaluated as well as the tissues’ viability and the ability to establish MSC cultures.

The application of PFLs for this procedure could improve the usability of pUCs as a valuable stem cell source, offering a promising alternative to antibiotics for decontamination purposes. pUCs were chosen because they offer a valuable source of MSCs without the need for invasive tissue sections [23,24]. In addition, due to their cylindrical geometry, they are complex in form, and microbial contamination may be difficult to reach. Therefore, it seemed plausible and promising to use plasma-processed liquids in a rinsing procedure to decontaminate the UCs. A successful realization will promote and advance the field of cultured meat production and will reduce reliance on antibiotics.

## 2. Results

### 2.1. Plasma Chemical Processes

The three plasma sources used in this study, namely wINPlas, wINPlas XXL, and flow DBD, have unique configurations but share similar reaction pathways that contribute to their antimicrobial efficacy. Understanding these pathways is crucial for optimizing their use in decontaminating porcine umbilical cords (pUCs). The wINPlas and wINPlas XXL devices both employ spark discharge to generate plasma at atmospheric pressure. The flow DBD system generates plasma through DBD in a flowing liquid environment, producing high concentrations of reactive species. This setup facilitates efficient gas–liquid interactions and continuous treatment of the liquid. In all three plasma sources, the primary reactions involve electron impacts where electrons collide with oxygen and water molecules, producing O_2_^−^ and OH. These reactive species undergo secondary reactions to form H_2_O_2_, NO_2_^−^, and NO_3_^−^. Additionally, O_3_ is produced through the reaction of oxygen molecules with atomic oxygen (O), generated by electron impact. The reactions listed below can be categorized based on the interaction phases in which they occur, namely gas-phase interactions, gas–liquid interface interactions, and liquid-phase interactions [5,25,26].

The plasma initiates electron impact reactions leading to the recombination of gas-phase species:

(1) $$N_{2}+O\to NO+N$$

(2) N+OH→NO+H

(3) $$NO+O/OH/HO_{2}\to NO_{2}$$

Gas–liquid interface:

(4) $$H_{2}O+e\to OH+H+e$$

(5) $$H+O_{2}\to HO_{2}$$

(6) $$OH+OH\to H_{2}O_{2}$$

(7) $$OH+NO\to HNO_{2}$$

(8) $$OH+NO_{2}\to HNO_{3}$$

Liquid phase species:

(9) $$H_{2}O_{2}+H_{3}O^{+}\to H_{3}O_{2}^{+}$$

(10) $$H_{3}O_{2}^{+}+HNO_{2}\to ONOOH+H_{3}O^{+}$$

(11) $$H_{2}O+NO_{2}+NO_{2}\to HNO_{2}+NO_{3}^{-}+H^{+}$$

(12) $$HNO_{2}+OH\to NO_{2}+H_{2}O$$

The reaction rates for HNO₂ and HNO₃ formation vary significantly depending on the plasma source used. In the wINPlas XXL system, HNO₂ forms at a rate of approximately 0.0522 mM/min, while HNO₃ forms at 0.0166 mM/min. In contrast, the flow DBD system, in high-power mode, exhibits significantly higher formation rates for both HNO₂ (0.60 mM/min) and HNO₃ (1.73 mM/min).

### 2.2. Physicochemical Characteristics

Table 1 compares the conditions and outcomes for producing PFLs using different plasma sources: wINPlas, wINPlas XXL, and flow DBD.

For the wINPlas device, which utilizes spark discharge with four pins, the PFL production condition was set at 0.5 L for 30 min. This system operates indirectly at temperatures exceeding 50 °C, resulting in a significantly low pH value of 2.35 and a high conductivity of 16,230 µS/cm. The conductivity of the untreated 0.9% NaCl solution was approximately 14,500 µS/cm. These results indicate substantial ionization and the production of reactive species.

The wINPlas XXL, also utilizing spark discharge but with 20 pins, was tested under various conditions: 6 L for 10, 20, 30, and 60 min, at room temperature. The indirect treatment showed a gradual decrease in pH with longer treatment times: 4.52 after 10 min, 3.35 after 20 min, 3.04 after 30 min, and 2.63 after 60 min (Table 1). The conductivity after 60 min was recorded at 16,725.70 µS/cm. This system handles larger volumes effectively, with increased acidification over time.

The flow DBD system, utilizing dielectric barrier discharge, was tested under three different conditions: 3 L for 30 min (indirect treatment at <40 °C, high power), 0.5 L for 15 min (indirect treatment at <50 °C, high power), and 0.5 L for 30 min (direct treatment at <40 °C, low power). The indirect treatment for 30 min resulted in a pH of 3.50 and conductivity of 16,597.30 µS/cm. The 15 min indirect treatment showed a pH of 3.47 and conductivity of 16,286.80 µS/cm, while the 30 min direct treatment yielded a pH of 3.31 and conductivity of 17,323.00 µS/cm. The flow DBD system offers versatile treatment modes, maintaining controlled temperatures and consistent ionization efficiency.

In summary, each plasma source has distinct operating conditions influencing the final pH and conductivity of the PFL. The wINPlas system operates at higher temperatures with significant acidification, the wINPlas XXL handles larger volumes with gradual pH reduction over time, while the flow DBD system offers flexible treatment modes with controlled temperatures and consistent ionization efficiency.

### 2.3. Change in Temperature and pH during Plasma Processing

Table 2 highlights reactive species production by the three plasma sources over various treatment times. The wINPlas device shows negligible H_2_O_2_ production but significant levels of NO_2_^−^ and NO_3_^−^ after 30 min, with concentrations of 1.83 ± 0.10 mM for NO_2_^−^ and 0.93 ± 0.09 mM for NO_3_^−^. The wINPlas XXL also shows negligible H_2_O_2_ but increasing concentrations of NO_2_^−^ and NO_3_^−^ over time. For instance, after 60 min of treatment, the concentrations are 2.70 ± 0.15 mM for NO_2_^−^ and 1.03 ± 0.10 mM for NO_3_^−^. For shorter treatment times of 10, 20, and 30 min, the NO_2_^−^ concentrations are 0.09 ± 0.005 mM, 0.90 ± 0.05 mM, and 1.35 ± 0.08 mM, respectively, while NO_3_^−^ concentrations are 0.20 ± 0.019 mM, 0.34 ± 0.03 mM, and 0.52 ± 0.05 mM, respectively.

The flow DBD system, both in high- and low-power settings, produces substantial amounts of H_2_O_2_, NO_2_^−^, and NO_3_^−^. Under high-power conditions, after 15 min of treatment, the concentrations are 5.00 ± 0.25 mM for H_2_O_2_, 10.00 ± 0.75 mM for NO_2_^−^, and 27.00 ± 1.0 mM for NO_3_^−^. After 30 min of high-power treatment, the concentrations increase to 8.00 ± 0.50 mM for H_2_O_2_, 19.00 ± 1.50 mM for NO_2_^−^, and 53.00 ± 2.0 mM for NO_3_^−^. Even under low-power conditions, the flow DBD system produces significant reactive species after 30 min, with concentrations of 5.60 ± 0.35 mM for H_2_O_2_, 13. ± 1.03 mM for NO_2_^−^, and 31.00 ± 1.17 mM for NO_3_^−^. These values demonstrate the higher efficacy of the flow DBD system in generating reactive species compared to the spark discharge systems. To achieve our aim of efficient decontamination of pUCs while maintaining cell viability, careful consideration of certain limiting factors is required, such as high temperature and very low pH, which vary depending on the plasma source and operation mode. As a result, the treatment time and operating conditions were selected based on these critical parameters.

### 2.4. Antimicrobial Activity

PFLs were produced by the three described plasma devices and used as washing liquid to decontaminate pUC tissue. Depending on the plasma device, two different procedures were followed. The liquid was either produced and then exposed to the tissue (indirect treatment mode), or the tissue was present during the plasma operation (direct treatment mode). The antimicrobial efficacy of the PFLs was assessed by plating samples of the washing liquid on agar plates and counting the colony-forming units (CFUs) after overnight incubation. In a second step, tissue samples were placed on agar plates before and after washing to assess the bacterial load on the tissue itself.

**Figure 1:**
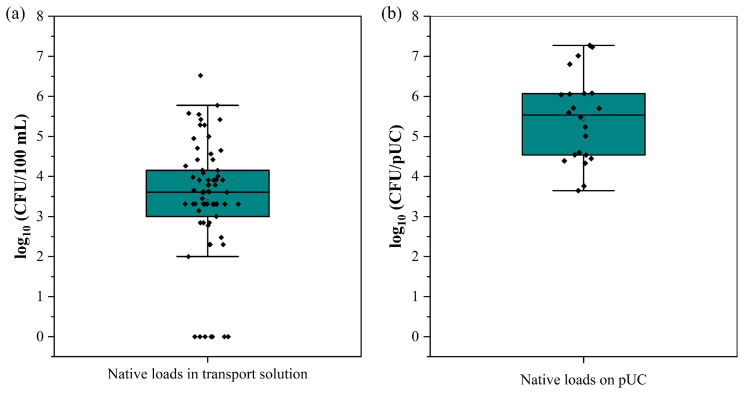
Microbial content in the transport solution (**a**) for collected umbilical cords and on the tissue samples (**b**) that provided the natural baseline contamination level. Box plots show mean with whiskers (25% and 75%) for n = 69 samples of transport solution and n = 22 samples for UC tissue.

Initially, the baseline contamination was determined in the solution in which tissue samples were transported (transport solution) and on the tissue itself. As shown in Figure 1, there is a large variation in bacterial concentrations of the original samples. This was evident in both the transport solution (Figure 1a) and on the tissue samples (Figure 1b).

The original bacterial loads of the 0.9% NaCl transport solution in which pUCs were collected ranged from a maximum of 5.78 to a minimum of 2.00 log CFU/100 mL, with a mean of 3.39 log CFU/100 mL (25–75% interval 3.00–4.15). By comparison, the total initial contamination of the pUC was also directly assessed on n = 22 samples, as shown in Figure 1b. The baseline contamination level of the tissue ranged from a maximum of 7.27 to a minimum of 3.65, with a mean value of 5.43 log CFU/pUC (25–75% interval 4.54–6.07). These values represent a substantial variation in the degree of contamination among the individual sampling days. Interestingly, the contamination level in the transport solution was significantly lower than on the umbilical cords, indicating that many microorganisms remained on or in the umbilical cords. Although it is possible to obtain pUC samples with low amounts of bacterial contamination, the presence of varying and occasionally high levels of natural contamination is beneficial for analyzing different decontamination scenarios using PFLs, thereby expanding the scope of decontamination strategies.

#### 2.4.1. Bacterial Species Identification

In addition to the quantitative analysis, we examined the quality of microbial contamination present on the tissue samples prior to intervention. Ten different strains of culturable bacteria were identified on the pUC samples (Table 3). Among them are predominantly Gram-positive (G^+^) bacteria and only three strains of Gram-negative (G^−^) bacteria. The identified species include *Escherichia coli*, *Aerococcus viridans*, *Streptococcus alactolyticus*, *Alloiococcus otitidis*, *Micrococcus luteus*, *Kocuria rhizophila*, *Acinetobacter pseudolwoffii*, *Corynebacterium xerosis*, *Corynebacterium* species, and *Psychroacter pulmonis*. These bacteria represent common environmental and opportunistic pathogens, with notable members such as *E. coli* and *Acinetobacter pseudolwoffii*, which are known for their resilience and potential to cause infections. The presence of these diverse microbial species underscores the importance of effective decontamination strategies for pUCs to ensure their suitability as a cell source for cell-based meat production.

#### 2.4.2. Antimicrobial Activity of Liquids Processed with wINPlas and wINPlas XXL

**Figure 2:**
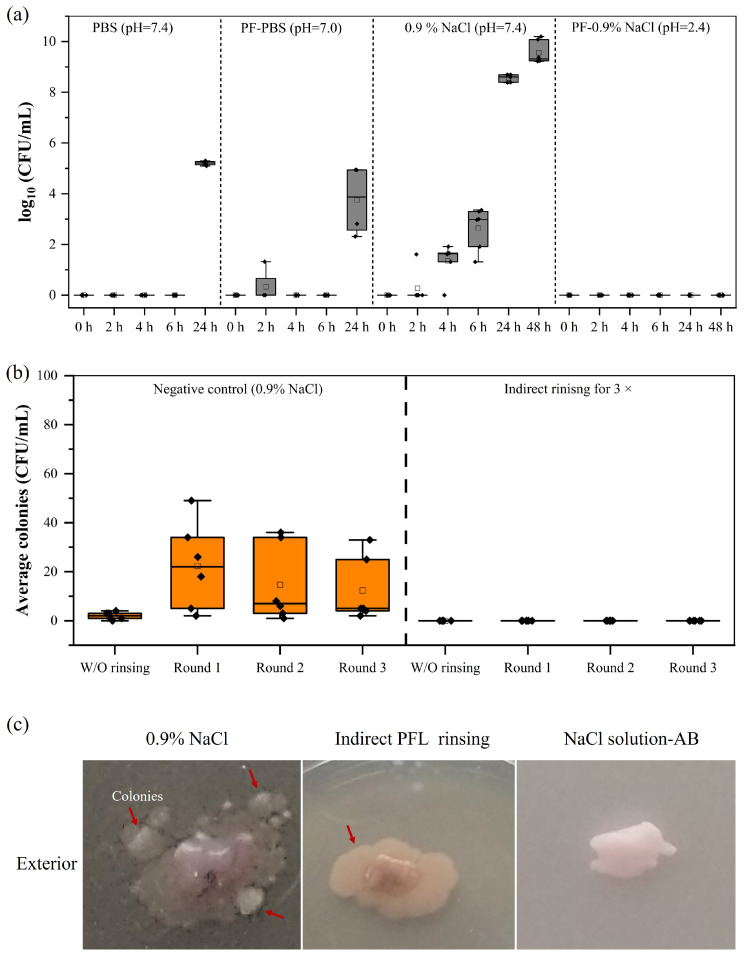
Bacterial content in PBS and 0.9% NaCl solution used as washing liquid for pUCs. (**a**) The wINPlas plasma source was operated for 30 min to process the liquid in which tissue samples were subsequently incubated for 2, 4, 6, 24, and 48 h in comparison to untreated solutions. Changes in pH value were monitored. (**b**) PF-0.9% NaCl solution was used for rinsing during 1–3 washing steps (rounds 1, 2, 3), and bacterial content decreased in comparison to untreated 0.9% NaCl. (**c**) Representative images of pUC tissue samples after three rinsing steps incubated on agar plates for 48 h. Bacterial colonies are indicated by red arrows. NaCl solution with antibiotics served as control. CFU: colony-forming units; PT-PBS and PT-0.9% NaCl: plasma-treated solutions.

To identify a suitable washing liquid for this application, it was necessary to maintain the tissue’s viability. Phosphate-buffered saline (PBS) and unbuffered physiological sodium chloride solution (0.9% NaCl) were employed for the washing procedure. Both liquids were processed in the wINPlas for 30 min prior to being used as washing solutions of the UC tissues. UC samples were incubated in PBS, plasma-treated PBS (PT-PBS), 0.9% NaCl solution, and plasma-treated 0.9% NaCl solution (PT-0.9% NaCl) for up to 24 and 48 h at room temperature. During this time, the tissues in untreated PBS and 0.9% NaCl solution retained their original tissue color, with visible traces of blood. When the tissue samples were incubated in plasma-treated liquids (PT-PBS and PT-0.9% NaCl), the tissue’s appearance changed to a whitish color. As the incubation time increased, both controls (untreated PBS and 0.9% NaCl solution) became turbid, indicating significant bacterial growth, amounting to more than 5.20 ± 0.09 log CFU/mL and 8.56 ± 0.14 log CFU/mL, respectively, after 24 h. After shorter exposure times of the liquid, the tissue samples retained their appearance, but CFUs could still be detected (Figure 2a).

Compared to PT-0.9% NaCl solution, PT-PBS gradually lost its antibacterial effect. Although almost no CFUs were detected in plasma-treated PBS for up to 6 h, the antibacterial effect on tissues was lost after 24 h. However, when UCs were incubated in plasma-treated 0.9% NaCl solution, no detectable CFUs were found even after 48 h. Consequently, 0.9% NaCl was used for all the following experiments.

The PFL affected the color and appearance of the UC tissue, indicating impaired tissue integrity. Therefore, it was investigated whether shorter repeated rinsing steps (rounds 1, 2, 3; Figure 2b) could be applied with the same efficacy in bacterial removal. The results revealed a bacterial reduction to the level of detection (Figure 2b) for PFL. The next step involved incubating tissue samples of pUCs on CASO-agar plates to detect any possible remaining bacteria on the tissue samples. As presented in Figure 2c, colony formation occurred when tissue was washed with just 0.9% NaCl solution but also in samples washed with PFL, indicating that decontamination was insufficient. 0.9% NaCl containing antibiotics was included as a positive control where no bacterial growth was detected.

**Figure 3:**
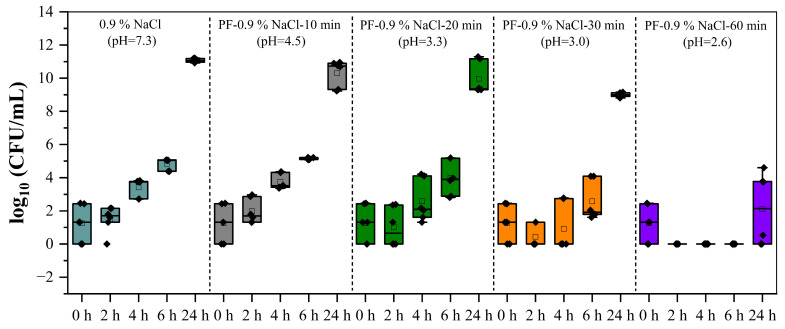
Bacterial content in wINPlas XXL treated 0.9%NaCl solution. Bacterial growth was detected in all samples after different plasma operation times of 10, 20, 30, and 60 min and following exposure times of 2, 4, 6, and 24 h. CFU: colony-forming units.

The following results pertain to the wINPlas XXL, which represents an upscaled version of the wINPlas. For the experiments, a total of 6 L of 0.9% NaCl was processed. During the operation, aliquots were withdrawn every 10 min for one hour, i.e., 10 min, 20 min, 30 min, and 60 min, and pUC samples were incubated in them. The results are shown in Figure 3. With increasing storage time from 0 to 24 h, the bacterial concentration in untreated 0.9% NaCl gradually increased (from 1.25 ± 1.09 log CFU/mL to 11.07 ± 0.11 log CFU/mL) (Figure 3). In freshly prepared PT-0.9% NaCl-10 min, a significant and continuous increase was observed over 24 h (up to 10.30 ± 0.81 log CFU/mL). pUCs incubated in freshly prepared PT-0.9% NaCl-20 or 30 min showed a reduction in bacterial content after 2 h. However, with increasing storage time from 2 to 24 h, the inactivation or antimicrobial efficiency was no longer evident, and the colonies amounted to 9.96 ± 0.98 and 8.99 ± 0.13 log CFU/mL, respectively. However, no detectable CFUs could be found for up to 6 h of incubation for the longest treatment time (PT-0.9% NaCl-60 min). After 24 h, a lower but significant increase in bacterial concentration was observed, reaching up to 2.11 ± 2.15 log CFU/mL. This indicates that even the longest plasma operation time tested (PT-0.9% NaCl-60 min) could not maintain its antibacterial effect over 24 h and was therefore excluded for further use in our application.

#### 2.4.3. Antimicrobial Activity of Flow DBD

**Figure 4:**
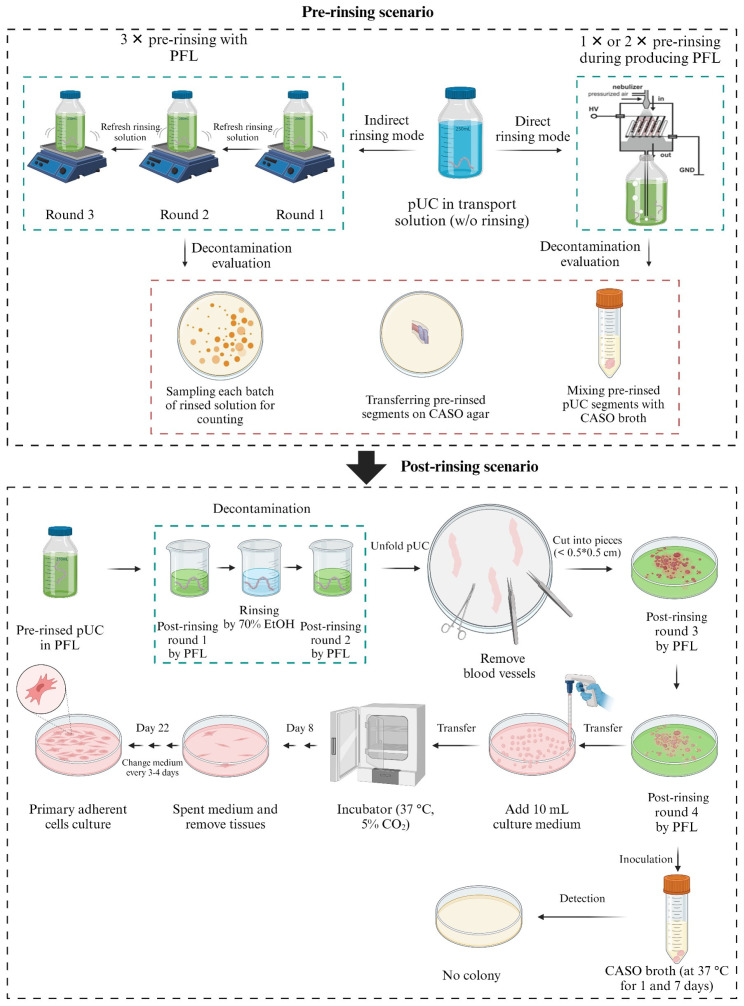
Workflow of the entire preparation of flow DBD-mediated PFL. The scheme includes a pre-rinsing scenario (top box) during which direct and indirect rinsing modes were tested. During the post-rinsing scenario (bottom box), additional rinsing steps were included until finally the tissue was transferred to an explant culture for the isolation of MSC.

The flow DBD system allows generating PFL, which can be used as a rinsing solution (indirect rinsing), or tissue samples can be directly exposed during the plasma operation (direct rinsing), as outlined in the rinsing procedures (Section 4.5). Both modes were used, and Figure 4 schematically summarizes the workflow.

##### Flow DBD-Mediated Indirect Treatment

**Figure 5:**
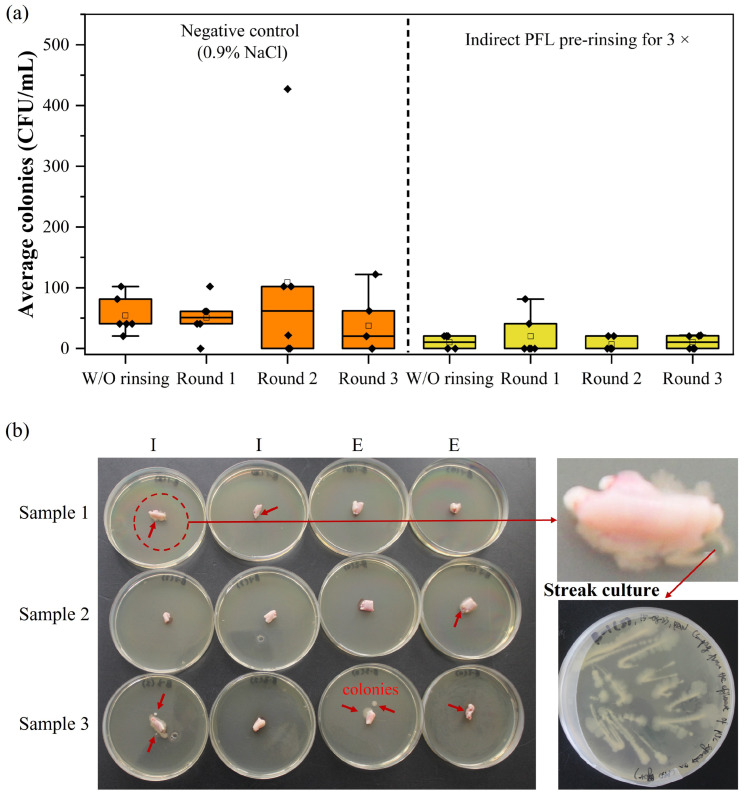
Bacterial content after rinsing with flow DBD-mediated PFL. (**a**) Tissue samples of pUC were washed in an indirect mode, and bacterial colonies were detected in the liquid in each of the 3 washes, i.e., rounds 1, 2, 3. (**b**) This was confirmed by bacterial growth from tissue samples on agar plates (after round 3). Red arrows indicate bacterial colonies. Magnification shows clearly the diffuse rim of the pUC tissue sample that resulted in smear colonies after streak culture. I: interior side of pUC, E: exterior side of pUC.

Next, we used the flow DBD plasma setting as described above. In total, 3 L of 0.9% NaCl solution was flushed through the system for 15 min (high power). Immediately afterwards, the liquid was applied to the pUC samples in several washing steps. The bacterial load was assessed in the liquid as well as on the tissue to detect any remaining bacteria. Both the liquid and the tissue yielded bacterial growth, as shown in Figure 5a,b. During the three washing steps, the bacterial content could be reduced in the liquid but not completely eliminated. When tissue samples were incubated on agar plates, a clear bacterial smear was observed as well (Figure 5b), indicating insufficient decontamination of the tissue surface.

##### Flow DBD-Mediated Direct Treatment

**Figure 6:**
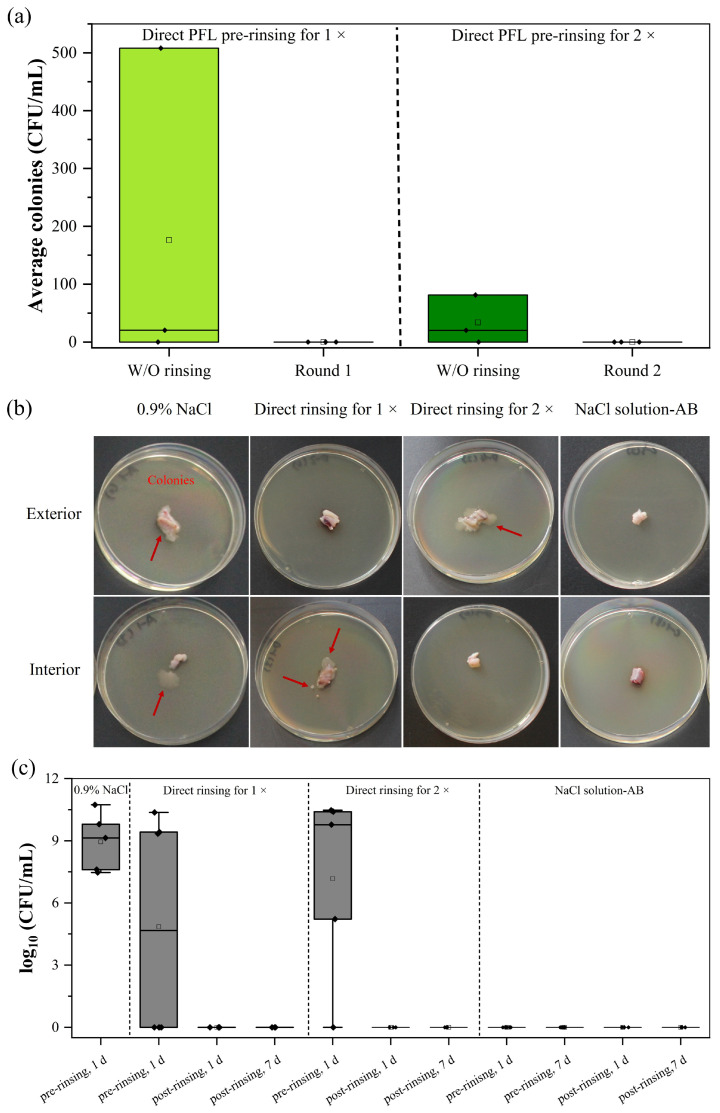
Bacterial content after rinsing in a direct treatment mode with the flow DBD. (**a**) Reduced number of bacterial colonies in the PFL after plasma processing once (round 1) and twice (round 2). (**b**) Segments of pUC (~1 cm) that were pre-rinsed with flow DBD-mediated PFL were placed on CASO-agar plate for 48 h (I: interior; E: exterior). Red arrows indicate bacterial growth. (**c**) The box plot represents the microbial load (log_10_ CFU/mL) observed after additional post-rinsing steps were included and tissue samples were incubated in liquid CASO broth for 1 day and 7 days (post-rinsing, 1 d and post-rinsing, 7 d). NaCl solution-AB (with antibiotic) served as positive control.

To improve the outcome and more efficiently remove bacterial contamination on the tissue samples, pUCs in liquid were exposed directly to the plasma discharge of the flow DBD system. Afterward, the rinsing liquid, as well as the tissue samples, were assessed for their bacterial content. Compared to the results from 0.9% NaCl and indirect PTL rinsing, the direct application of PTL for 1× or 2× was more effective in reducing the bacterial load in the rinsing solution (Figure 6a). Figure 6b confirms that persistent bacterial colonies remained on the tissue segments, although no bacteria were detectable in the rinsing solution after two rounds of plasma operation (round 1, round 2; Figure 6b). This indicates that the bacteria were not washed off but rather adhered to the tissue, while samples treated with antibiotic-containing solution revealed no bacterial growth. A second rinse did not significantly improve the decontamination efficacy compared to a single rinse (Figure 6b). Considering the remaining persistent microorganisms, the pieces of UC tissue were further rinsed (post-rinsing, compare Figure 4) and finally incubated in liquid CASO broth to cultivate any remaining bacteria. As presented in Figure 6c, this additional rinsing procedure removed any remaining bacteria from the tissue. After day 1 and day 7 in liquid CASO broth, no bacterial colonies were detected. This finding confirms that with additional rinsing steps, an effective decontamination of pUC can be achieved by the flow DBD system.

### 2.5. Investigation on Tissue Viability

**Figure 7:**
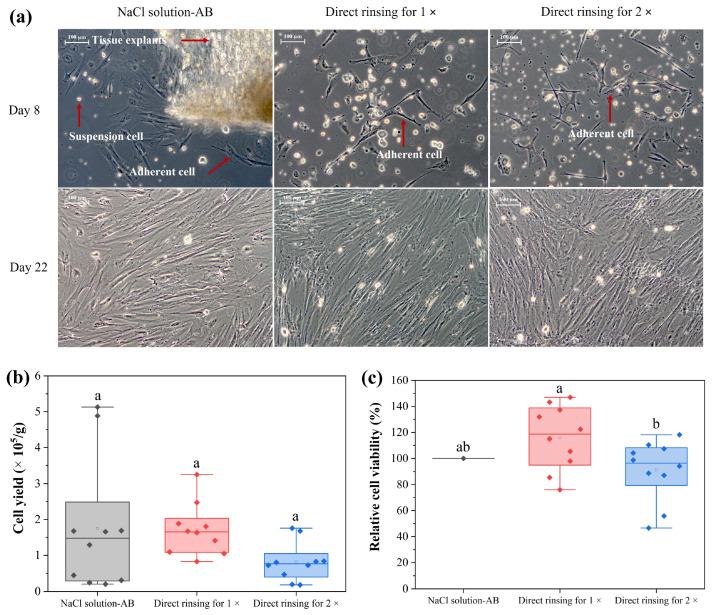
Microscopic images from tissue explants (**a**) and resulting cell yield (**b**) and cell viability (**c**) of isolated cells. (**a**) Pieces of pUC were used as explants to isolate MSCs (magnification ×100). Left: primary cells isolated from explants rinsed with antibiotic-containing solution at day 8 and 22 of cell culture; middle, right: primary cells isolated from pUC after rinsing with flow DBD-mediated PFL 1× as well as for rinsing 2× at day 8 and day 22. (**b**) Cell yield per g of tissue at day 22 of cell culture. (**c**) Relative cell viability at day 22 of cell culture. Values result from 5 biological replicates with 2 technical replicates. Different letters indicate significant differences (*p* < 0.05) between samples. Scale bar: 100 µm.

After complete decontamination of the UC by PFL, the intactness of the treated tissue was investigated. We used pieces of pUC in an explant culture approach to gain viable, proliferating cells. Figure 7 illustrates microscopic images of an explant culture after 8 days and 22 days. After 8 days, there are a few elongated, spindle-shaped cells and many loose cells that have not yet attached. At day 22, however, cells have proliferated and present a confluent cell layer of MSCs (Figure 7). This result indicates that treatment with PFL (flow DBD plus rinsing steps) preserves the integrity of the pUC tissue and maintains the cell’s viability.

As shown in Figure 7b, the final cell yield from tissues rinsed with NaCl solution with antibiotics (positive control), direct PFL 1× and 2× were 1.75 × 10^5^ cells/g (±1.82), 1.71 × 10^5^ cells/g (±0.72), and 8.19 × 10^4^ cells/g (±5.34), respectively. A one-way analysis of variance revealed no significant difference between the three categories (*p* > 0.05), indicating that tissue rinsing with PFL in a direct treatment mode 1× or 2× had no negative effect on the final cell yield of the explant culture. There was a slightly reduced cell viability in all three samples, as presented in Figure 7c. The cell viability after rinsing with NaCl solution-AB (positive control), direct rinsing 1× and 2× was 40.23% (±7.63), 46.73% (±9.87), and 36.66% (±9.34), respectively. There is no significant difference between the NaCl solution-AB and PFL rinsing 1× (*p* > 0.05) as well as between the NaCl solution-AB and PFL rinsing 2× (*p* > 0.05), while there is a significant difference between PFL rinsing 1× and 2× (*p* < 0.05). This finding indicates that a direct rinsing in PFL 1× was superior to 2× regarding cell viability.

## 3. Discussion

Plasma-functionalized liquids (PFLs) are being explored for a range of applications in the field of plasma medicine and life sciences [14,27,28,29]. Due to their physicochemical properties, PFLs possess antimicrobial properties that may replace chemical sanitizing solutions for food and agricultural produce, including salad, seeds, meat, and others [12,14,30,31,32]. Along this line, we aimed to investigate the efficacy of PFLs for the decontamination of animal tissue samples by different plasma-generating devices. Porcine umbilical cords (pUCs) were used as naturally contaminated tissue because they provide an ethically acceptable cell source for the recovery of pluripotent stem cells with the prospect of further development into cell-based meat products [23,24,33]. Prior to the isolation of stem cells, a thorough decontamination process is required to provide clean source material.

In this study, we employed three different plasma devices to generate PFLs that yielded different antimicrobial efficacies. For each plasma device, the procedure needed to be adapted depending on the parameters and the volume of liquid (Table 1). Physicochemical analyses of the liquids revealed differences in the plasma-generated species that could explain the observed variations. The bacteria remaining after different washing procedures were analyzed, and conclusions concerning antimicrobial efficacy were drawn. Although the microbial content of the washing liquid could be reduced below the limit of detection, remaining bacteria were still detected on the tissue samples. Several washing steps were necessary to eliminate microorganisms completely. Finally, the explant culture method was used to isolate viable and proliferating mesenchymal stem cells (MSCs) from PFL-exposed pUCs that proved the integrity of the tissue.

Our results demonstrate that PFLs generated by spark discharge (wINPlas, wINPlas XXL) and flow DBD effectively decontaminate pUCs, with flow DBD and wINPlas showing the highest efficacy. Various plasma sources have been used for generating PFLs with the purpose of eradicating microorganisms, for instance gliding arc [34], corona discharge, plasma jet [35], spark discharge [36], and DBD [3]. They all have in common that they produce RONS as soon as they come in contact with air and the air–liquid interphase [37]. However, the concentration of generated species varies among the plasma systems and hence in the resulting treated liquids. The chemical analysis confirms that the plasma sources used in this approach produce sufficient reactive oxygen and nitrogen species, measured as H_2_O_2_, NO_2_^−^, and NO_3_^−^, to ensure microbial inactivation. The lowest concentrations were found in the four-pin spark discharge (wINPlas), while higher concentrations were measured in the flow DBD system. Especially H_2_O_2_ was only generated in the flow DBD, while it was neglectable in the spark discharge. The synergistic effects of nitrites and hydrogen peroxides in combination with an acidic pH determine the antimicrobial activity [5,38].

pH levels were carefully monitored in our experiments, and a reduced pH was detected for all tested devices (Table 2). The most investigated solutions for antimicrobial efficacy are water, saline, and phosphate-buffered saline [39,40]. In terms of changes in pH and the generation of RONS, both water and saline achieved similar outcomes, leading to comparable inactivation rates. In our study, we excluded water because maintaining the tissue’s viability was crucial. Buffered saline and saline differed greatly in inactivation efficacy, with better reduction rates in the saline solution (Figure 4). This underlines the role of pH, as acidification of liquid during plasma treatment promotes the conversion to nitrous acid, which is an essential step in the antimicrobial process and in good agreement with previous reports on several microorganisms [4,35,41].

Moreover, the importance of maintaining pH levels above 2.0 to prevent tissue damage while achieving effective decontamination is supported by our results, which emphasized the critical balance between antimicrobial activity and tissue preservation. Conversely, the flow DBD system, while maintaining a higher pH level, is capable of generating significantly higher concentrations of reactive species, including O_3_, H_2_O_2_, NO_2_^−^, and NO_3_^−^ (Table 3). These high levels of reactive species in the DBD system ensure robust microbial inactivation, even if the pH level is not as low as that produced by the wINPlas systems. Our findings further corroborate the superior performance of plasma sources that produce higher concentrations of ozone and other ROS and RNS. The difference in H_2_O_2_ production between the wINPlas and the flow DBD systems can be attributed to the distinct mechanisms and conditions under which these plasma systems operate. The wINPlas system generates plasma through a spark discharge mechanism. Spark discharges typically create high-energy electrons that ionize the gas and liquid molecules in their vicinity. This environment favors the formation of other reactive species, such as O_3_ and nitric oxides (NOx), over hydrogen peroxide. The intense and localized energy input in spark discharges leads to reaction pathways that are less favorable for H_2_O_2_ formation.

In contrast, the flow DBD system generates plasma through dielectric barrier discharge, which produces a more diffuse and uniform plasma. This setup facilitates the production of RONS, including H_2_O_2_. The conditions in DBD plasmas, such as lower electron temperature and more homogeneous discharge, are more conducive to the formation of H_2_O_2_ compared to spark discharges. In our measurements, H₂O₂ was detected using UV/VIS spectroscopy. Notably, these were endpoint measurements, and they capture the final concentration of species after all reactions had occurred. It is important to note that during the plasma treatment, degradation reactions of H₂O₂ also take place, reducing its concentration over time. In fact, the concentration of H₂O₂ detected was so low that it fell within the measurement uncertainty, making it difficult to distinguish from background noise. This indicates that H₂O₂ degradation was likely significant, contributing to the overall low levels observed, which align with previous reports [36,42]. Additionally, gas–liquid interaction plays a significant role. The spark discharge system in wINPlas often has less efficient gas–liquid interaction compared to DBD systems. In contrast, the flow DBD system ensures better mixing and interaction between the plasma and the liquid. The continuous flow allows for more uniform treatment and efficient transfer of reactive species into the liquid, which reflects in the different reaction rates. Operational conditions also contribute to the differences. The wINPlas system operates at high power and frequency, leading to high local temperatures that can decompose H_2_O_2_ if formed. Conversely, the flow DBD system generally operates under less extreme conditions, with lower local temperatures that favor the stability and accumulation of H_2_O_2_ in the treated liquid. The difference in temperature levels between the plasma systems also plays a crucial role in their effectiveness. The flow DBD system, while producing higher concentrations of reactive species, tends to have a slight temperature increase, which must be carefully managed to avoid compromising the integrity of heat-sensitive tissue, such as pUCs. The flow DBD system’s configuration allows for better dissipation of heat due to the continuous flow of liquid through the discharge region, which helps in maintaining lower temperatures compared to static systems like wINPlas. Excessive heat can not only damage the tissue but can also permanently harm cell functionality. Therefore, maintaining the temperature within safe ranges is essential, limiting the treatment time in direct applications. Balancing the generation of reactive species with controlled temperature levels is critical for optimizing the decontamination process while preserving tissue viability.

For future industrial applications of PFLs, scalability remains a challenge [30,33]. This concerns on one hand the generation of PFLs and on the other hand the cell yield. The existing literature has demonstrated the scalability of microwave-driven plasma sources for generating large volumes of PFLs with high concentrations of reactive species, which have effectively reduced microbial loads in treated liquids and on salad [31,43]. In our study, the wINPlas XXL represents an upscaled version of the wINPlas, which is capable of generating larger volumes and higher concentrations of RONS, predominantly NO_2_^−^, and NO_3_^−^, enhancing its efficacy in the decontamination process. When using the flow DBD system, we were able to generate 6 L of PFLs with even higher concentration of RONS (Table 3) and better antimicrobial efficacy.

Regarding the cell yield, our results demonstrate that we are able to isolate MSCs that are proliferating, as can be seen in a confluent cell layer after 22 days in culture (Figure 7). However, much higher cell yields are necessary to proceed with cultivation in bioreactors for tissue formation. An estimated 4 × 10^7^ cells/mL are required to grow 1000 kg of cultured meat in a bioreactor of 20.000 L [44].

A major advantage of using PFL is that it facilitates the antimicrobial effects on internal tissue structures that are otherwise difficult to reach, such as the inside of the cylindric structure of pUCs. Adherent microbial contaminants were still detectable on the inner tissue surface, although the washing liquid was free of bacteria. Therefore, it was necessary to cut open the tissue and add more washing steps with PFLs to eliminate bacteria completely (post-rinsing step). Our bacterial growth methods were adapted, and even the most sensitive method of incubating tissue samples in liquid agar broth finally showed no detectable colonies anymore (Figure 6c).

Decontaminating umbilical cords poses unique challenges due to their complex structure and organic composition. The presence of blood vessels and connective tissue can harbor microbes and protect them from decontaminating agents. Previous studies have shown that the presence of organic material reduced the antimicrobial efficacy of plasma-treated liquids significantly [45]. In our hands, the best results were achieved when PFL of a flow DBD was used and several washing steps were included. The highest concentrations of RONS were generated by the flow DBD, and with each renewal of PFL, fresh RONS were applied to the tissue. In addition, it should be considered that we used UCs with their natural bacterial contaminants (Figure 3, Table 4), which contain significantly lower concentrations of CFU than in experimental setups (6–9 × 10^6^ CFU/mL) [45]. Moreover, most bacteria reside on the outside of the pUC and are primarily targeted, while MSCs are well protected inside the tissue where RONS may not directly reach. Therefore, MSCs could well be recovered in reasonable cell yields.

The results obtained in this study highlight the varying efficacies of different plasma sources, particularly comparing the DBD system with the wINPlas and wINPlas XXL systems. One of the critical differences between these systems lies in the pH levels of the PFLs they produce. The pH levels of the PFLs generated by the wINPlas systems were significantly lower compared to those produced by the DBD system, which plays a vital role in the inactivation of microorganisms.

The combination of a highly acidic environment and the presence of RONS in the wINPlas systems provides a synergistic effect that enhances the antimicrobial efficacy. However, the DBD system compensates for its higher pH with the sheer concentration of reactive species it generates, providing a superior effective decontamination performance. Both approaches have their distinct advantages, and understanding these differences is crucial for optimizing the decontamination processes for this application.

Our findings support the potential of using PFL as a sustainable and effective alternative to antibiotics for decontaminating pUCs without interfering with the tissue integrity in the production of cultured meat. Further research is needed to improve the cell yield and explore the long-term effects of PFLs on stem cell viability and differentiation potential.

## 4. Materials and Methods

### 4.1. Plasma-Generating Systems

#### 4.1.1. Spark Discharge Devices: wINPlas and wINPlas XXL

**Figure 8:**
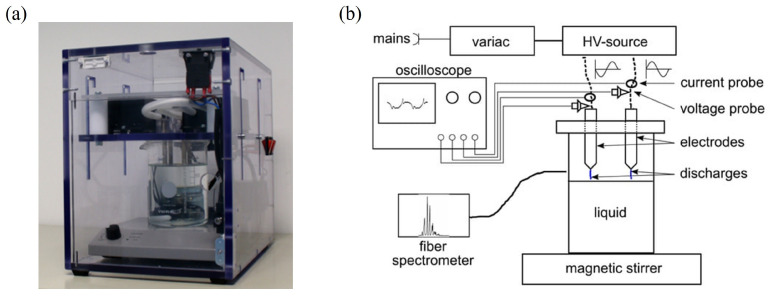
Photograph of the spark discharge device inside a plexiglass box (wINPlas) (**a**) and a scheme of it (**b**). The power supply and the positioning of the electrodes on top of the liquid are indicated. Adapted from [36].

The wINPlas device is an atmospheric pressure plasma system designed for small-scale applications (Figure 8). The compact reactor allows precise and localized plasma treatment of liquids for volumes of up to 500 mL, making it ideal for laboratory-scale experiments. It employs a spark discharge configuration to generate plasma within a confined space. Key components include a high-voltage power supply and four electrodes. When an alternating current (AC) is applied, spark discharges occur, producing plasma on the surface of a liquid. It operates at a frequency of 25 kHz and 50 W power [36].

The wINPlas XXL is an upscaled version of the wINPlas device, designed to handle larger liquid volumes for industrial-scale applications up to 6 L. It maintains the fundamental spark discharge mechanism but with enhanced capabilities. Plasma generation is realized by spark discharge, ensuring consistent and effective liquid treatment. The device operates at a frequency of 50/60 Hz with adjustable power levels between 66 and 72 W. The plasma source consists of 10 ignition transformers (COTI TRK2-35, Treviso, Italy) operating with a total of 20 stainless steel metal pins. Ignition transformers activate the plasma discharges, creating a spark discharge between the pins and the surface of the flowing liquid with pins positioned approximately 2 mm above the liquid surface. A continuous flow process allows the liquid to be supplied continuously at 25 L/h through the plasma source. The operating parameters, including frequency, power, and discharge gap between the pin bottom and the liquid surface are set at 50/60 Hz, 66–72 W, and 2 mm, respectively, ensuring effective plasma generation. This setup allows the wINPlas XXL to effectively treat larger volumes of liquid, making it suitable for high-throughput industrial applications while maintaining the required consistency and effectiveness in plasma treatment.

#### 4.1.2. Flow DBD

**Figure 9:**
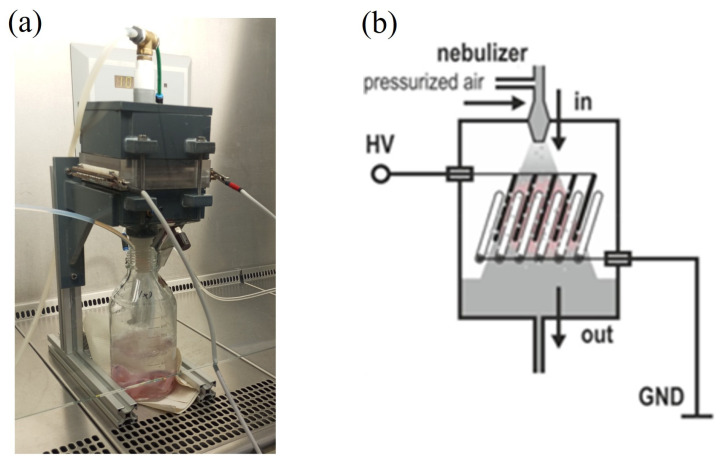
Photograph of the flow DBD (**a**) and a scheme of the electrodes and flow direction (**b**). HV: high-voltage electrode, GND: grounded electrode. Adapted from [46].

The flow DBD system is designed to generate PFL through direct discharge in a flowing liquid environment. In this context, the term “flow DBD” refers to a dielectric barrier discharge system specifically designed for processing liquids by incorporating a liquid circulation system. Although the system can function without liquids, it has been adapted for applications where liquid treatment is essential. This setup is highly efficient in producing reactive species and is versatile in various applications [18]. The flow DBD system (Figure 9) generates dielectric barrier discharges in humid air containing water droplets by providing negative high-voltage pulses with a duration of 100–550 ns and a negative amplitude of −10.7 kV from a pulse generator (Eagle Harbor NSP-120-20-N, Seattle, WA, USA) at a repetition rate of 1.5 kHz. The plasma source comprises 14 parallel tungsten electrodes, each 120 mm in length and 2 mm in diameter, spaced 6 mm apart. Another layer of 15 electrodes is arranged similarly below but offset horizontally by 3 mm. The electrodes in the lower layer are embedded in quartz tubes and are grounded. The chamber containing the water mist measures 120 × 120 mm at the base and is 200 mm in height [46].

### 4.2. Preparation of Plasma-Functionalized Liquid (PFL)

Three different plasma sources were used to generate PFLs for the rinsing experiments: two spark discharge systems (wINPlas and wINPlas XXL) and one flow-through dielectric barrier discharge.

PFLs were prepared by treating sterile PBS and 0.9% NaCl solutions, respectively. PBS was only used with the wINPlas to initially compare antimicrobial effects. For all other experiments, a 0.9% NaCl solution was used. The treatment conditions, including power settings and exposure times, were optimized to maximize the generation of reactive oxygen and nitrogen species (RONS) detected as H_2_O_2_, O_3_, NO_2_^−^, and NO_3_^−^, and they are summarized in Table 1.

### 4.3. Physicochemical Measurements

After plasma treatment, the pH value, conductivity, and temperature of the liquid were measured using a multiparameter meter (Hanna Instruments HI 9828, Vöhringen, Germany). Chemical components in the form of nitrogen species, specifically NO_2_^−^ and NO_3_^−^, were detected by ion chromatography using a Dionex ICS 5000 system (Thermo Scientific, Dreieich, Germany) with UV and conductivity detectors. The concentration of H_2_O_2_ in the PFL was determined using UV absorption spectroscopy from the focused illumination by a Deuterium lamp (DH-2000 Ocean Optics, Duiven, The Netherlands). The spectra were recorded on a fiber-optic spectrometer (AvaSpec Dual, Apeldoorn, The Netherlands).

For the treatment, different volumes and times were applied depending on the plasma source (Table 1). For wINPlas, 500 mL of PBS or 0.9% NaCl solution was treated for 30 min. The wINPlas XXL device treated up to 6 L of 0.9% NaCl solution in a continuous flow process at a flow rate of 25 L/h, with treatment times of 10, 20, 30, and 60 min. The flow DBD system treated 3 L of 0.9% NaCl solution for 30 min at high power and 500 mL for 30 min at low power. High-power settings for the flow DBD were defined as a pulse width of coarse 500 µs and fine 50 µs with a pulse repetition frequency of base 2 kHz and multiplier 1.5, while low-power settings were a pulse width of coarse 100 µs and fine 0 µs with the same pulse repetition frequency.

### 4.4. Sample Collection and Transport

pUCs were kindly provided by the Institute for Muscle Biology and Growth, Research Institute for Farm Animal Biology (FBN; Dummerstorf, Germany). The specimens were transported to the Leibniz Institute for Plasma Science and Technology (INP) in 100 mL of either sterile 0.9% NaCl (*w*/*v*) solution or PFL, or NaCl solution with antibiotics (0.9% NaCl with 10% penicillin/streptomycin and 10% amphotericin B), all kept on ice. To minimize waste and consider animal welfare, overly long pUCs were divided into 2–3 samples and allocated across various PFL rinsing scenarios.

In the initial experiments with the wINPlas and wINPlas XXL, the pH of the NaCl-solution was adjusted to the pH of PBS (around 7.4) by adding 0.1 mol/L NaOH solution or 1 mol/L HCl. However, this practice was discontinued once PBS was excluded as a treatment solution due to unsatisfactory antimicrobiological results. It was decided not to adjust the pH in subsequent experiments to avoid any potential negative impacts on the antimicrobial efficacy of the NaCl solution. This approach ensured that the observed effects were solely due to the plasma treatment without interference from other agents.

### 4.5. Rinsing Procedures

The pUC samples underwent rinsing procedures either as an indirect or a direct rinsing procedure, as applicable (Table 1). The rinsing step primarily targeted the exterior surface of the entire pUC. For indirect rinsing, PFL was generated first and then used to rinse the pUC (scenario 2a and 2b). The entire pUC was transferred into a 250 mL sterile Schott bottle and pre-rinsed with 100 mL PFL while shaking at 150 rpm for 10 min (Grant-Bio ES-80 Shaker-Incubator, Cambridge, UK).

In direct rinsing, liquid containing the pUC was directly exposed to the plasma process. Due to temperature sensitivity (below 40 °C) and toxicity concerns, only the flow DBD system was suitable for direct rinsing (scenario 3a and 3b).

As a positive control, an antibiotic solution (NaCl solution-AB) was included (scenario 4). This contained 10% penicillin/streptomycin (Pan Biotech™, Aidenbach, Germany), 10% amphotericin B (Pan Biotech™, Aidenbach, Germany) in 0.9% NaCl. Untreated solutions served as negative controls (scenario 1).

For the flow DBD system, an additional post-rinsing procedure was introduced. In this procedure, the pUC samples were subjected to a second rinse with freshly generated PFL after the initial plasma treatment. This step aimed to enhance the decontamination efficiency by ensuring that any remaining microorganisms on the interior and exterior surfaces of the pUC segments were effectively inactivated. This two-step process—pre-rinsing followed by post-rinsing—provided a comprehensive decontamination strategy and is an integral part of the explant culture procedure.

### 4.6. Microbial Analysis

After adjusting the PFL to the ambient temperature, pUCs were placed in sterile Schott bottles containing 30 mL of PFL. The pUCs were exposed to PFL for 0, 2, 4, 6, 24, and 48 h at 37 °C with constant agitation at 50 rpm in an incubator. After each exposure interval, 50 µL of each sample was inoculated onto soybean-casein digest agar (CASO-agar, Merck, Darmstadt, Germany) plates (9 cm diameter) using a spiral plater (Eddy Jet 2, IUL, Barcelona, Spain). The bacterial concentration in the original transport medium was also measured. For samples stored for 24 h with microbial loads exceeding 6 log CFUs/mL, gradient dilution was performed to obtain countable colony numbers. The CASO-agar plates were incubated at 37 °C for 24 h. CFUs/mL were determined using the spiral plate method and quantified with an automated colony counter (Flash & Go, IUL, Barcelona, Spain).

After the rinsing stage, approximately 1 cm sections from both ends of the pUC were removed and discarded. The remaining samples were uniformly cut into fragments measuring 0.5–1 cm. To evaluate the decontamination effectiveness of PFL rinsing on both the interior and exterior surfaces of the pUC, the cords were slit longitudinally. Fragments were then randomly placed onto CASO-agar plates with either side facing down and incubated at 37 °C for 48 h. The evaluation included three biological replicates, with four technical replicates each (two for interior and two for exterior surfaces).

In addition to the agar plate method, a more sensitive culture condition in CASO broth was conducted for the flow DBD system to assess microbial growth in a liquid medium. pUC fragments were placed in 50 mL Falcon tubes containing 20 mL of CASO broth (Merck, Darmstadt, Germany) and incubated at 37 °C for 1 to 7 days. After each incubation period, 50 µL of the broth was sampled and inoculated onto CASO-agar plates using the spiral plater. The plates were then incubated at 37 °C for 18–24 h to check for microbial growth. The method has a sensitivity of 1 CFU/mL.

Experiments were performed in triplicate with parallel samples. NaCl solution without plasma processing served as a control.

In addition, native culturable aerobic bacterial strains on pUCs were isolated and identified by an external service laboratory (IMD, Greifswald, Germany).

### 4.7. Creating Explants

To create the explant culture, umbilical cords were cut longitudinally, and blood vessels were removed. Afterwards, several washing steps followed, as illustrated in Figure 4 (post-rinsing). Finally, each segment was cut into 0.5 × 0.5 cm pieces and transferred into a petri dish with around 15 mL of PFL for a washing step. For the positive control (scenario 4, Table 1), every post-rinsing step was replaced by NaCl solution-AB instead of using PFL (scenario 3a and 3b). Pieces of pUC were placed in a cell culture dish (Ø 10 cm, Sarstedt, Nümbrecht, Germany) with the inner side facing down. Excess liquid was removed before carefully adding 10 mL of explant culture medium (alpha MEM supplemented with 10% FCS and 1% penicillin/streptomycin and 1% amphotericin B (all PAN-Biotech GmbH, Aidenbach, Germany)) into each dish. Incubation was performed at 37 °C with 5% CO_2_. On day 8, the tissue pieces were removed, and the culture was continued with 10 mL of culture medium. Fresh culture medium was added every third day until day 22. During the culture period, images of growing MSC cells were obtained using the Zeiss Axiovert 40 CFL microscope (Carl Zeiss, Göttingen, Germany) with a 10× magnification objective coupled to a high-resolution Axiocam-40 CFL digital camera (model Axiocam MRC) for morphological characterization.

On day 22, the medium was aspirated, and cells were washed twice with 1× DPBS (devoid of calcium and magnesium; PAN-Biotech GmbH, Aidenbach, Germany). Cells were covered with 5 mL of Accutase^®^ enzyme (Accutase Cell Detachment Solution, BioLegend, San Diego, CA, USA) and allowed to detach at 37 °C for 15–20 min, followed by a gentle rinse with 1 mL of DPBS. Subsequently, the cells in suspension were centrifuged at 500 rpm for 8 min, the supernatant was removed, and the cell pellet was resuspended in 1 mL of explant medium (alpha MEM, supplemented with 10% FCS, 1% penicillin/streptomycin, and 1% amphotericin B). A CytoFLEX-S flow cytometer (Beckman Coulter, Brea, CA, USA) was used to determine the number of cells and their viability after mixing 20 μL of the cell suspension with 180 μL of DAPI (10 μM, Sigma-Aldrich, Taufkirchen, Germany).

### 4.8. Statistical Analysis

A one-way analysis of variance (ANOVA) was performed to test for significant differences in cell yield and cell viability across the different treatment groups. This method compares the means of multiple groups to determine if there are any statistically significant differences between them by analyzing the variance within each group and between groups.

## 5. Conclusions

In conclusion, this study successfully demonstrated that plasma-functionalized liquids can achieve effective decontamination of porcine umbilical cords while preserving cell viability, a critical factor for subsequent cell culture applications. Direct rinsing methods in particular proved significantly more effective than indirect approaches, allowing for deeper and more thorough decontamination of tissue surfaces. Among the plasma systems tested, the flow DBD system consistently outperformed others, showing higher efficiency in generating reactive species and maintaining stable pH conditions, which are essential for both microbial inactivation and cell preservation. The results emphasize that the efficacy of decontamination is closely tied to the plasma configuration used. These findings establish a strong foundation for utilizing plasma-based decontamination in cultured meat production, offering an innovative, antibiotic-free approach to ensure tissue sterility without compromising cell health.

## Figures and Tables

**Table 1:** Comparison of technical parameters of wINPlas, wINPlas XXL, and flow DBD for the production of PFLs.

Plasma Source	Discharge Type	Condition for Producing PFL		Indirect or Direct Treatment	Temperature (°C)	Final pH Value	Conductivity (µS/cm)
**wINPlas**	spark (4 pins)	0.5 L	30 min	indirect	>50 °C	2.35	16,230.0
**wINPlas XXL**	spark (20 pins)	6 L	10 min	indirect	RT	4.52	
			20 min	indirect	RT	3.35	
			30 min	indirect	RT	3.04	
			60 min	indirect	RT	2.63	16,725.7
**flow DBD**	DBD	3 L	30 min	indirect	<40 °C	3.5	16,597.3
			high power				
		0.5 L	15 min	indirect	<50 °C	3.47	16,286.8
			high power				
		0.5 L	30 min	direct	<40 °C	3.31	17,323.0
			low power				

**Table 2:** Chemical composition of liquids after plasma processing with wINPlas, wINPlas XXL, and flow DBD. H_2_O_2_, NO_2_^−^, and NO_3_^−^ were determined as stable species representative for reactive oxygen and nitrogen species (RONS). Intensity of gray scale indicates increased concentrations. Data are mean and SD of three experiments.

	**Spark Discharge**	**Spark Discharge XXL**				**Flow DBD**		
	Treatment Time [min]	Treatment Time [min]				Treatment Time [min]		
**Reactive Species**	30	10	20	30	60	15	30	30
						high power	high power	low power
H_2_O_2_ (mM)	negl.	negl.	negl.	negl.	negl.	5.00	8.00	5.60
						±0.25	±0.5	±0.35
NO_2_^−^ (mM)	1.83	0.09	0.90	1.35	2.70	10.00	19.00	13.00
	±0.10	±0.005	±0.05	±0.08	±0.15	±0.75	±1.5	±1.03
NO_3_^−^ (mM)	0.93	0.2	0.34	0.52	1.03	27.00	53.00	31.00
	±0.09	±0.019	±0.03	±0.05	±0.10	±1.0	±2.0	±1.17

**Table 3:** Summary of the identified bacterial species on the umbilical cord tissue in order of frequency of detection.

Identified Species	Gram Strain	Note
*Escherichia coli*	*G^−^*	
*Aerococcus viridans*	*G^+^*	*Streptococcus* family
*Streptococcus alactolyticus*	*G^+^*	*Streptococcus* family
*Alloiococcus otitidis*	*G^+^*	
*Micrococcus luteus*	*G^+^*	*Micrococcus* family
*Kocuria rhizophila*	*G^+^*	*Micrococcus* family
*Acinetobacter pseudolwoffii*	*G^−^*	
*Corynebacterium xerosis*	*G^+^*	*Corynebacteriaceae* family
*Corynebacterium species*	*G^+^*	*Corynebacteriaceae* family
*Psychroacter pulmonis*	*G^−^*	

**Table 4:** Overview of experimental conditions used for different decontamination rinsing scenarios.

Scenario	Plasma Source	Treatment	Transport Solution		Pre-Rinsing				Post-Rinsing		
			Solution	Volume	Solution	Volume	Time	Rounds	Solution	Volume	Rounds
1	-	Negative control	0.9% NaCl	200 mL	0.9% NaCl	100 mL	10 min	3×	-	-	-
2a	wINPlas	Indirect rinsing	0.9% NaCl/PFL	200 mL	PFL	100 mL	10 min	3×	-	-	-
2b	Flow DBD	Indirect rinsing	PFL	100 mL	PFL	100 mL	10 min	3×	-	-	-
3a		Direct rinsing 1×	0.9% NaCl	100 mL	PFL	500 mL	30 min	1×	PFL	20 mL	4×
3b		Direct rinsing 2×	0.9% NaCl	100 mL	PFL	500 mL	30 min	2×	PFL	20 mL	4×
2c	wINPlas	Indirect rinsing	0.9% NaCl	200 mL	PFL	30 mL	24 h	1×	-	-	-
2d	wINPlas XXL	Indirect rinsing	0.9% NaCl	200 mL	PFL	30 mL	24 h	1×	-	-	-
4	˗	Positive control	0.9% NaCl-AB	100 mL	+AB	100 mL	10 min	3×	+AB	20 mL	4×

## Data Availability

The data that support the findings of this study are available on request from the corresponding author, [S.H.].
